# Supplementary material for: Accuracy of Anti-SARS-CoV-2 Antibody in Comparison with Surrogate Viral Neutralization Test in Persons Living with HIV, Systemic Lupus Erythematosus, and Chronic Kidney Disease
Source: Vaccines (Basel). 2024 May 20;12(5):558. doi: 10.3390/vaccines12050558 (PMC11125822; doi:10.3390/vaccines12050558)
Supplement: Supplementary file 1 [file vaccines-12-00558-s001.zip › vaccines-2965532-supplementary.pdf]

Table S1. Table of subjects characteritics, anti-RBD and sVNT results based on age group

	**Young Adult (18-39) N = 248**	**Middle Age (40-59) N = 218**	**Elderly (60+) N = 51**	**p**
**Gender, n (%)**				
Male	124 (50)	91 (41.74)	24 (47.06)	ns
Female	124 (50)	127 (58.26)	27 (52.94)	
**History of COVID-19, n (%)**				
No	199 (80.24)	48 (22.00)	35 (68.63)	ns
Yes	49 (19.76)	170 (78.00)	16 (31.37)	
**Vaccination, n (%)**				
Vaccinated	134 (54.00)	136 (62.39)	31 (60.78)	ns
Unvaccinated	114 (45.00)	82 (37.61)	20 (39.22)	
**Vaccine Type (%)**				
CoronaVac	96 (71.64)	73 (53.68)	16 (51.61)	
mRNA	36 (26.87)	62 (45.59)	15 (48.39)	0.0134
ChAdOx1-S	2 (1.49)	1 (0.73)	0 (0)	
**Result**				
**Anti RBD (AU), Median (IQR)**				
Unvaccinated	8.8 (0.4 – 62.3)	17.2 (0.8 – 113.2)	68.3 (3.3 – 119.5)	ns
Vaccinated	95.7 (13.8 – 201)	160.3 (18.5 – 200)	74.04 (10.5 – 200)	ns
**sVNT (% inhibition), Median (IQR)**				
Unvaccinated	29.1 (4.4 – 71.9)	48.1 (9.6 – 92.6)	86.9 (19.6 – 94.7)	0.0066
Vaccinated	82.6 (34.6 – 94.6)	91.1 (41 – 95.6)	86.7 (22.9 – 95.6)	ns
